# Transcriptomic Analysis in Human Macrophages Infected with Therapeutic Failure Clinical Isolates of *Leishmania infantum*

**DOI:** 10.1021/acsinfecdis.1c00513

**Published:** 2022-03-30

**Authors:** Ana Perea-Martínez, Raquel García-Hernández, José Ignacio Manzano, Francisco Gamarro

**Affiliations:** Instituto de Parasitología y Biomedicina “López-Neyra”, IPBLN-CSIC, Parque Tecnológico de Ciencias de la Salud, Avda del Conocimiento 17, 18016 Armilla, Granada, Spain

**Keywords:** human macrophages, Leishmania infantum, therapeutic failure clinical parasites, infection, transcriptomic analysis, modulation of host cells

## Abstract

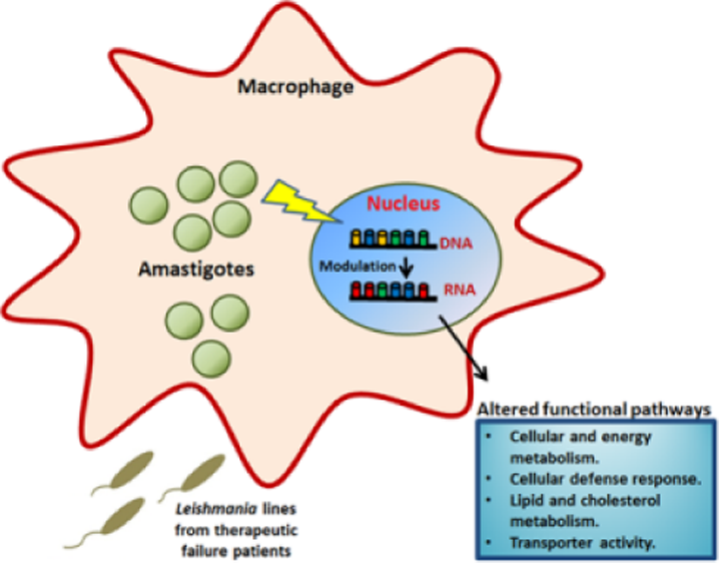

Leishmaniasis is one of the neglected tropical diseases with a worldwide distribution, affecting humans and animals. In the absence of an effective vaccine, current treatment is through the use of chemotherapy; however, existing treatments have frequent appearance of drug resistance and therapeutic failure (TF). The identification of factors that contribute to TF in leishmaniasis will provide the basis for a future therapeutic strategy more efficient for the control of this disease. In this article, we have evaluated the transcriptomic changes in the host cells THP-1 after infection with clinical *Leishmania infantum* isolates from leishmaniasis patients with TF. Our results show that distinct *L. infantum* isolates differentially modulate host cell response, inducing phenotypic changes that probably may account for parasite survival and TF of patients. Analysis of differential expression genes (DEGs), with a statistical significance threshold of a fold change ≥ 2 and a false discovery rate value ≤ 0.05, revealed a different number of DEGs according to the *Leishmania*line. Globally, there was a similar number of genes up- and downregulated in all the infected host THP-1 cells, with exception of Hi-L2221, which showed a higher number of downregulated DEGs. We observed a total of 58 DEGs commonly modulated in all infected host cells, including upregulated (log_2_FC ≥ 1) and downregulated (log_2_FC ≤ −1) genes. Based on the results obtained from the analysis of RNA-seq, volcano plot, and GO enrichment analysis, we identified the most significant transcripts of relevance for their possible contribution to the TF observed in patients with leishmaniasis.

Leishmaniasis is a neglected tropical disease caused by the protozoan parasite *Leishmania* that in their visceral form may result lethal if left untreated. It represents the second parasitic disease after malaria in the number of deaths, with a high mortality rate and an increase in the number of cases around the world due to globalization and climate change. Chemotherapy remains the only effective weapon against leishmaniasis; however, the arsenal of drugs in use is reduced and limited mainly to four drugs including amphotericin B (AmB), miltefosine (Mil), paromomycin (PMM), and antimonials (Sb^III^). The emergence of drug resistance and therapeutic failure (TF) impacts treatment outcome, and their understanding will be of help for new therapeutic strategies to control this relevant infectious disease. TF is a clinical phenotype of patients in whom clinical symptoms do not improve after drug treatment (non-response) or reappear after an initial cure (relapse). It has been accepted that in leishmaniasis, TF and drug resistance are not necessarily synonymous, being frequently confounded. However, it must be stated that drug resistance to a drug is only one of the possible factors that contribute to TF.

TF in parasitic diseases has a multifactorial origin, involving a considerable number of factors in the host (immunity or nutritional status), the parasite (drug resistance, infectivity, parasite localization and accessibility to drugs, coinfection with other pathogens), the drug (quality, pharmacokinetics), and the environment (global warming and the expansion of the disease to new geographical areas), influencing treatment outcomes.^1^

Additionally, host factors play an important role in TF through the requirement of an effective immune response to support anti-leishmanial drugs. It has been described that patients with immunodeficiency show difficulties for an effective cure.^2^ Natural variation of host immunological response and differences between patients can also influence the ability of drugs to efficiently work on intracellular parasites.^3−5^ It has been considered that several parasite capabilities such as infectivity and/or host manipulation might contribute to TF.^6^

In this regard, during host–pathogen interactions, it has been described that there are global changes in the gene expression pattern both in the host cells and in the infecting pathogens^7−9^ contributing to the parasite’s ability to evade host defenses mechanisms, including the production of reactive oxygen and nitrogen species, activity of proteases, and acidification, and to survive intracellularly, avoiding the toxic effects of anti-leishmanial drugs. Intracellular pathogens have evolved many strategies to counteract these defenses mediated by virulence factors, by a significant reprogramming of their host cells by modulation of signaling pathways and chromatin remodeling, or by mechanisms to evade autophagy.^10^ Transcriptomic analyses through the high-throughput deep sequencing (dual-RNA-Seq) technology have been implemented to understand how different *Leishmania* species^11−14^ or *Leishmania* parasites with different infectivity capacities^15,16^ modulated host response upon infection. Dual-RNAseq in *Leishmania*-infected host cells have demonstrated a response variable according to the period post infection (initial or established infection stages).^13,14,17,18^ Modulation of host response by intracellular *Leishmania* parasites has shown a differential expression genes (DEGs) ranging from a general gene repression^19,20^ to a significant gene over-expression in macrophages infected with *Leishmania* parasites after 24 h.^21^ Globally, all results suggest that macrophage gene expression modulation is dynamic and may be linked to the *Leishmania* infecting species and the stage of infection in which expression is evaluated.

The identification of factors that contribute to TF in leishmaniasis will provide the basis for a future and more efficient therapeutic strategy that will improve the control of this neglected disease. In this article, we study by RNAseq at a later time point of infection, the transcriptomic analysis of host THP-1 cells infected with clinical isolates of *Leishmania infantum* from TF patients with leishmaniasis. We described various important host factors modulated by these parasites which could be associated with the parasite’s ability to survive to drug activity in the host macrophages and probably could contribute to TF.

## Results and Discussion

### Drug Sensitivity Profile of *L. infantum* Lines

In the present work, we focus on the ability of different clinical isolates of *L. infantum* parasites from TF HIV patients with VL and unsuccessfully treated with liposomal AmB to modulate the gene expression of host cells at 96 h post infection. In this way, first, we analyzed the drug sensitivity profile of these *L. infantum* lines to the commonly used leishmanicidal drugs Sb^III^, PMM, Mil, and AmB. The results showed that all *L. infantum* lines in their promastigote forms were sensitive to the mentioned drugs. Focusing on the amastigote forms, we got the same results as observed for promastigotes, with the exception of L2221; this line was more than 1.6-fold resistant to Sb^III^ compared to the susceptible line LJPC, used as a control reference line (Table 1). Interestingly, the patient from whom this L2221 line was isolated was not treated with Sb^III^-based drugs. Consequently, considering the sensitivity of the different clinical isolates of *L. infantum* to anti-leishmanial drugs, the TF observed in patients could be due to other different factors such as modulation of host cellular functions by these parasites. Additionally, it should be taken into account that anti-leishmanial drugs show different effectiveness in the case of patients co-infected with HIV. For example, some authors have reported that Mil is less effective in men co-infected with HIV in comparison with sodium stibogluconate treatment.^22^ Certainly, drug resistance is not the unique aspect to be considered when studying TF as it can be caused by a plethora of reasons, such as increased infectivity, resistance of *Leishmania* to reactive oxygen species,^6^ or, as we hypothesize, alteration of gene expression of host cells promoted by these parasites.

**Table 1:** Drug Sensitivity Profile of Amastigote Forms of Clinical Isolates of *L. infantum*^a^

	EC_50_ (μM) ± SD [RI]^b^			
*L. infantum* line	AmB (μM)	Mil (μM)	PMM (μM)	Sb^III^ (μM)
LJPC	0.07 ± 0.01	1.46 ± 0.15	127.32 ± 3.32	44.66 ± 5.60
L2070	0.12 ± 0.02	1.21 ± 0.15	119.99 ± 26.54	48.08 ± 8.71
L2165	0.11 ± 0.02	2.40 ± 0.38	180.00 ± 11.66	31.10 ± 2.82
L2255	0.04 ± 0.01	1.52 ± 0.31	168.55 ± 29.39	41.56 ± 4.04
L2221	0.06 ± 0.01	2.11 ± 0.14	194.31 ± 17.11	>70 [>1.6]*

a Parasites were grown as described in the Methods section in the presence of increasing concentrations of compounds. The *L. infantum* LJPC line was used as a reference line.

b Resistant index (EC_50_ in *L. infantum* lines/EC_50_ in LJPC) is presented in brackets. Data are means ± standard deviations from three independent experiments. Significant differences were determined by using Student’s *t*-test (*p < 0.01); for sensitivity to Sb^III^ in the L2221 line, we used Student’s *t*-test (*n* = 3) with one right tail and 70 μM as the most restrictive value.

As the results of this work rely on the capacity of intracellular *Leishmania* parasites to modulate the host cells, it was necessary to check the infectivity of the different parasite lines. Thus, we established cell culture conditions to ensure high and equivalent levels of infection of the different *L. infantum* lines. Thus, THP-1 cells were infected with parasites in the stationary phase and infectivity profiles were analyzed at 96 h post infection. We observed a percentage of infection up to 76% in the *L. infantum* lines, with the number of amastigotes per cell up to 11. In conclusion, infectivity and the number of intracellular parasites were high enough for all *Leishmania* lines, allowing the detection of the gene expression modulation of host cells caused by these parasites.

Contrary to the majority of infection studies carried out to date in which early infection times were used,^11,15^ our study was made at 96 h in order to let the parasite settle inside the cells and give it the chance to modulate the gene expression of the host cells. In this way, we can obtain a pool of data including valuable information about how parasites that led to TF could increase their survival through alteration of the host cellular functions.

### Transcriptomic Profile of Host Infected Cells

We analyzed the transcriptomic profile of three independent biological replicates from THP-1 cells infected with different *L. infantum* lines obtained from TF patients (Hi-L2221, Hi-L2165, Hi-L2070, and Hi-L2255) and the reference line (Hi-LJPC) versus non-infected THP-1 cells. The unspecific transcripts associated with phagocytosis were subtracted from the transcriptomic analysis of host cells infected with parasites killed by heat shock as unspecific genes related with phagocytosis.

**Figure 1:**
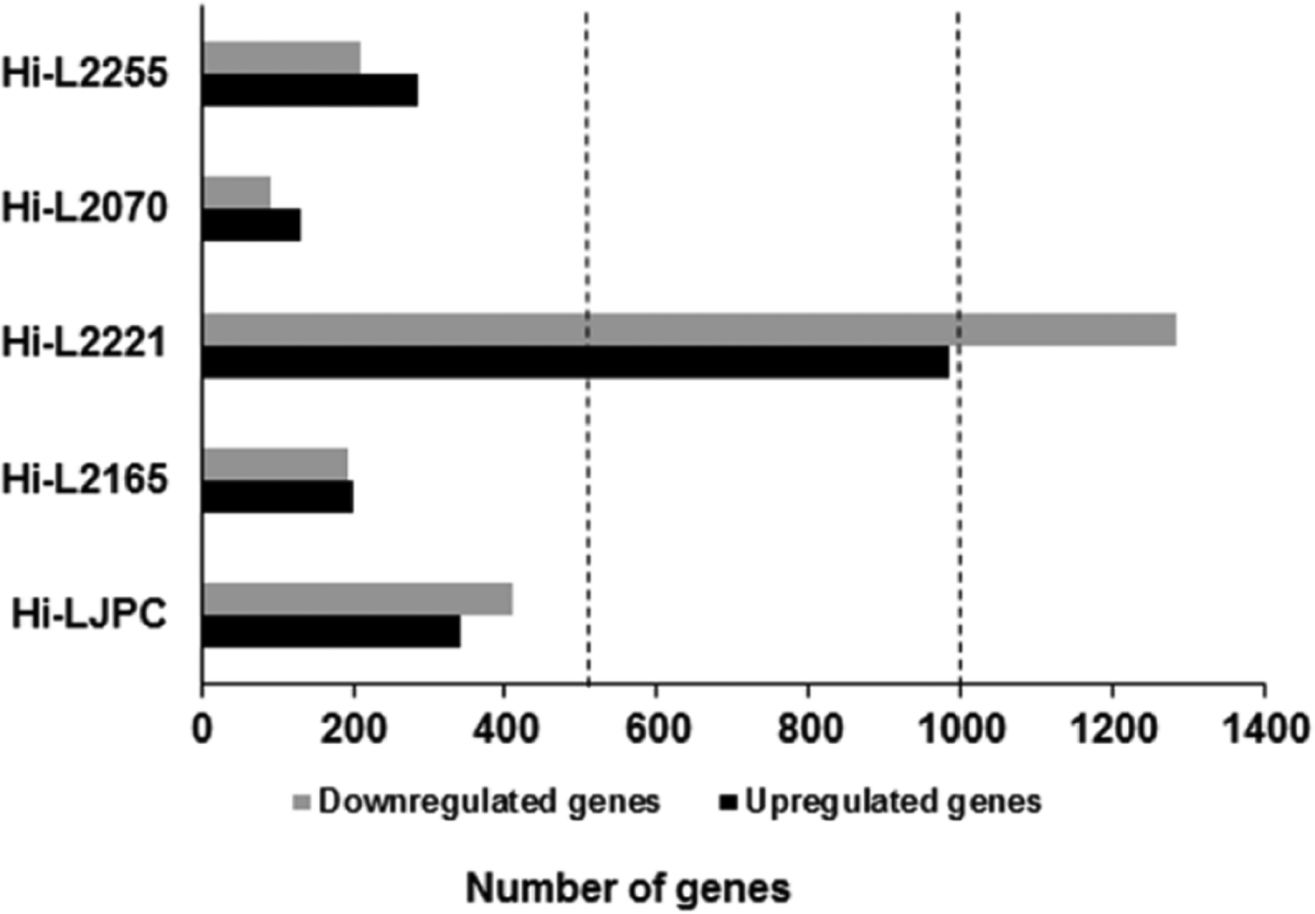
Transcriptome profiles of THP-1 cells infected with different *L. infantum* lines. Differential gene expression profiles are presented as the numbers of upregulated (black) and downregulated (dark gray) transcripts after comparisons of the different infected cell lines vs non-infected cells (the unspecific phagocytosis background has been subtracted previously). The data are from three independent biological replicates, considering a fold change ≥ 2 and an FDR value ≤ 0.05.

Analysis of DEGs with a statistical significance threshold of a fold change ≥ 2 and a false discovery rate (FDR) value ≤ 0.05 revealed different numbers of DEGs according to the *Leishmania* line; specifically, in Hi-LJPC, 341 genes were upregulated, and 411 genes were downregulated; comparison (non-infected cells subtracting non-specific DEGs of phagocytosis) versus Hi-L2221 revealed the highest amount of DEGs with 1044 upregulated genes and 1586 downregulated genes (Figure 1). In contrast, the rest of the comparisons showed a similar number of DEGs between them. Hi-L2070 was the sample with the lowest number of changes in expressed genes with only 132 upregulated genes and 89 downregulated genes, while Hi-L2165 comparison revealed 207 upregulated genes and 202 downregulated genes and Hi-L2255 with 306 and 218, respectively (Figure 1). Globally, there were a similar number of genes up- and downregulated in all the infected host THP-1 cells, with exception of Hi-L2221, which showed 4-fold more downregulated DEGs than the other three infected host cells with TF lines. After *Leishmania* infection, it has been described that there is a strong regulation of host gene expression, presenting a similar number of DEGs between the different *Leishmania* lines employed.^20,23^

This study agrees with the more recent reports showing similar numbers of genes induced and repressed following infection by clinical isolates of *L. infantum* lines, with the exception of Hi-L2221.^11,14,15^ An interesting observation was the high number of DEGs in Hi-L2221 versus the other infected host cells; these results could be due to a need for a strong modulation of the host cells by these parasites, allowing the survival to the stress of the intracellular environment and the escape from the defense of the immune system of patients contributing to the TF.

**Figure 2:**
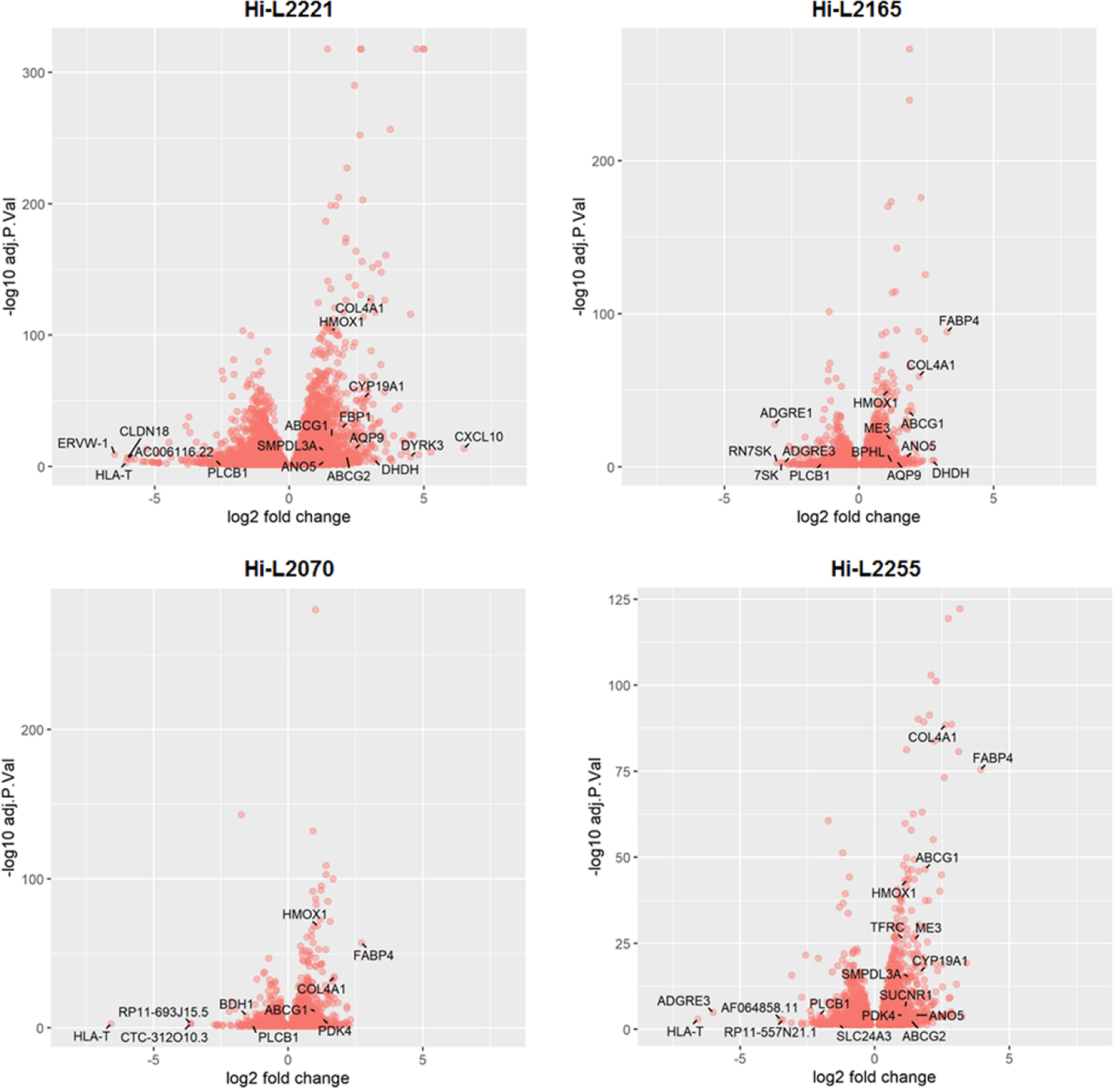
Volcano plots from RNAseq data of THP-1 cells infected with different *L. infantum* lines. The log_2_FC is plotted on the *x*-axis, and the negative log_10_FDR (adjusted *p*-value) is plotted on the *y*-axis. The represented genes have an FDR lower than 0.05. Some relevant DEGs are indicated with an arrow.

We generated volcano plots comparing the fold changes in expression (Log_2_) with the corresponding adjusted *p*-values (−log_10_) (Figure 2); the results show the gene expression profile subtracting the statistical non-significant genes of host cells infected with different clinical isolates of *L. infantum* lines. We indicated the DEGs with the highest Log_2_ fold change as well as the genes involved in the most relevant functions in the host cells (Figure 2).

**Figure 3:**
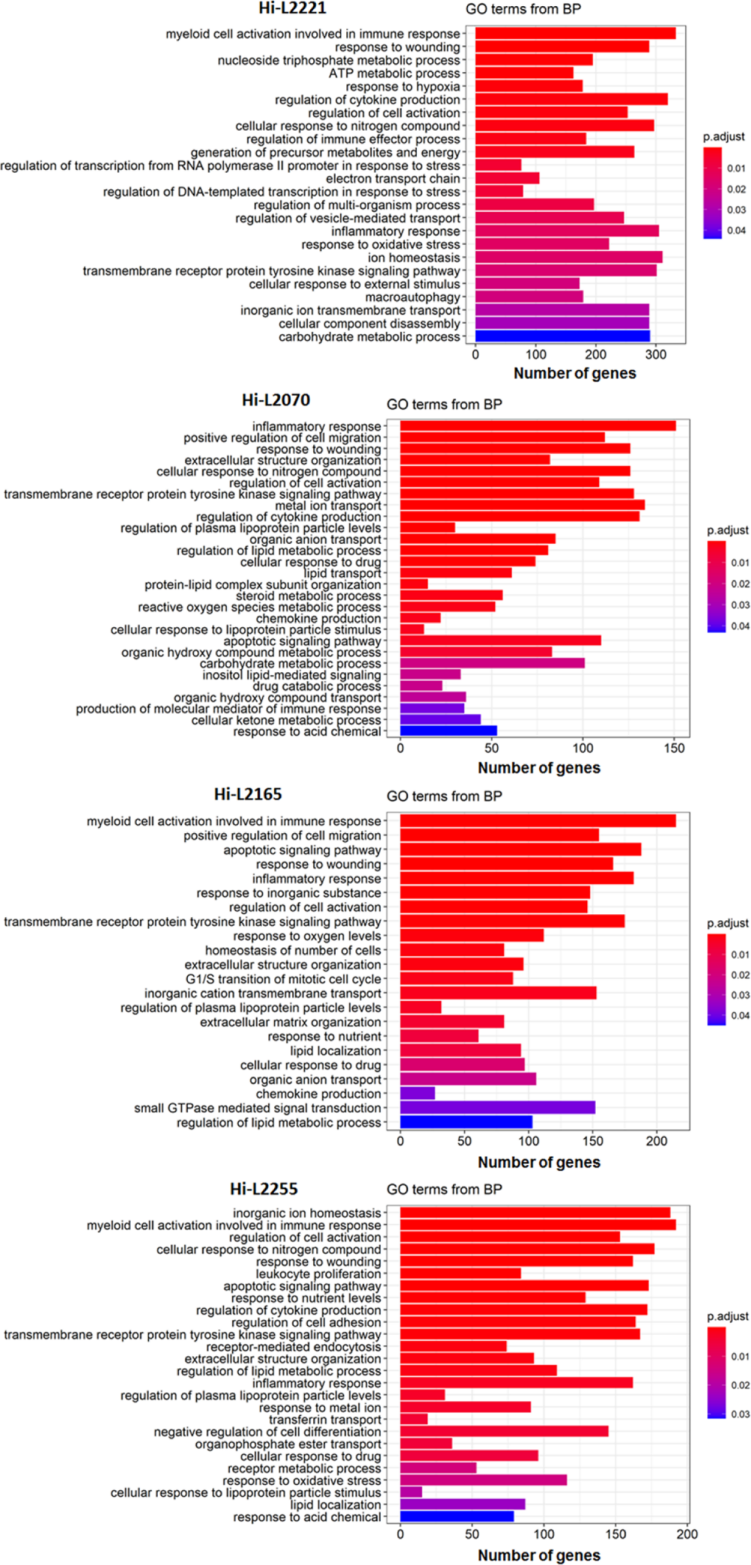
GO term enrichment analysis of DEGs including in the category Biological Processes. Enriched GO terms for most relevant DEGs of lines Hi-L2221, Hi-L2165, Hi-L2070 and Hi-L2255. The number of DEGs is expressed in the *x*-axis and colors represent the significance level (FDR) of the enrichment.

To obtain a deeper insight into the biological processes of up- and downregulated DEGs, we performed GO term enrichment analysis. In this work, we focused on DEGs associated with biological process categories mainly related to the ability of the parasite to evade the host cell defense mechanisms. Secondarily, we have studied categories involved in the metabolism, autophagy, transmembrane transport, and the lipid metabolism. According to the above, the significantly enriched routes that belong to the host immune system and present in most of the different lines were associated to processes as “inflammatory response” (305 DEGs in Hi-L2221, 182 DEGs in Hi-L2165, 162 in Hi-L2255, and 151 in Hi-L2070), “myeloid leukocyte mediated immunity” (333 DEGs in Hi-L2221, 215 DEGs in Hi-L2165, and 192 in Hi-L2255), “regulation of cytokine production” (320 DEGs in Hi-L2221, 172 in Hi-L2255, and 131 DEGs in Hi-L2070), “transmembrane receptor protein tyrosine kinase signaling pathway” (320 in Hi-L2221, 175 DEGs in Hi-L2165, 167 Hi-L2255, and 128 in Hi-L2070), and “response to wounding” (222 in Hi-L2221, 61 DEGs in Hi-L2165, 162 Hi-L2255, and 126 in Hi-L2070). In addition, other significantly enriched routes related with the immune system were “regulation of cell activation”, “regulation of immune effector process”, “chemokine production”, “cellular response to drug”, “cellular response to nitrogen compound”, and “leukocyte proliferation” (Figure 3). Finally, routes related with autophagy, solute transport, and the energetic and oxidative metabolism were found significantly enriched in the host cells infected with the different *Leishmania* lines: “carbohydrate metabolic process”, “regulation of the lipid metabolic process”, “response to oxidative stress”, “macroautophagy”, “ATP metabolic process”, “apoptotic signaling pathway“, “drug catabolic process”, “ion homeostasis”, “steroid metabolic process”, “lipid transport”, “transferrin transport”, “response to nutrient levels”, “regulation of vesicle-mediated transport”, “generation of precursor metabolites and energy”, “metabolic ion transport”, “organic anion transport” and “electron transport chain”, among others (Figure 3).

Based on the results obtained from the analysis of RNA-seq information and volcano plot and according to GO enrichment analysis of the DEGs, we identified in THP-1 cells infected with TF clinical *Leishmania* isolates the most relevant transcripts: *ABCA10*, *ABCG1*, *ABCG2*, *ANO5*, *AQP9*, *BDH1*, *BPHL*, *COL4A1*, *CYP19A1*, *DHDH*, *DYRK3*, *FBP1*, *HMOX1*, *ME3*, *PDK4*, *PLCB1*, *SLC24A3*, *SMPDL3A*, *SUCNR1* and *TFRC* (Table 2); these DEGs could contribute to TF in patients infected with these *L. infantum* lines.

**Table 2:** Profile of DEGs Most Relevant for the THP-1 Modulation after Infection with *L. infantum* Lines^a^

Gene ID	Description	Function	Hi-L	logFC	FDR value
*ABCG1*	ATP binding, cassette,sub-family G, (WHITE), member, 1	phospholipid efflux	Hi-L2165	1.84	5.36 × 10^–37^
			Hi-L2070	1.01	9.49 × 10^–12^
			Hi-L2221	1.58	2.22 × 10^–23^
			Hi-L2255	1.87	2.46 × 10^–47^
*COL4A1*	collagen, type, IV, alpha, 1	cellular response to the nitrogen compound	Hi-L2221	3.01	3.50 × 10^–129^
			Hi-L2165	2.22	9.67 × 10^–60^
			Hi-L2255	2.62	3.01 × 10^–89^
			Hi-L2070	1.71	3.26 × 10^–35^
*HMOX1*	heme, oxygenase, (decycling), 1	autophagy	Hi-L2165	1.08	1.20 × 10^–50^
			Hi-L2070	1.08	3.41 × 10^–69^
			Hi-L2221	1.56	8.32 × 10^–104^
			Hi-L2255	1.18	4.84 × 10^–44^
*PLCB1*	phospholipase, C, beta, 1, (phosphoinositide-specific)	regulation of multiorganism process	Hi-L2165	–1.39	0.00394942
			Hi-L2070	–1.33	0.00748531
			Hi-L2221	–2.73	1.31 × 10^–5^
			Hi-L2255	–2.05	6.76 × 10^–5^
*ANO5*	anoctamin 5	anion transport	Hi-L2165	1.76	1.03 × 10^–6^
			Hi-L2221	1.29	9.77 × 10^–5^
			Hi-L2255	1.50	8.51 × 10^–5^
*ABCG2*	ATP binding cassette, sub-family G (WHITE), member 2	cellular detoxification	Hi-L2221	2.12	8.78 × 10^–9^
			Hi-L2255	1.33	0.002619
*AQP9*	aquaporin, 9	cellular response to the organonitrogen compound	Hi-L2165	1.40	0.00030758
			Hi-L2221	2.43	6.95 × 10^–14^
*CYP19A1*	cytochrome P450, family 19,sub-family A, polypeptide 1	drug metabolism, sterol metabolic process, electron transport chain, lipid transport, and inflammatory response	Hi-L2221	2.78	4.09 × 10^–53^
			Hi-L2255	1.67	2.10 × 10^–17^
*DHDH*	dihydrodiol, dehydrogenase, (dimeric)	generation of precursor metabolites and energy and electron transport chain	Hi-L2165	2.72	9.58 × 10^–5^
			Hi-L2221	3.16	4.96 × 10^–6^
*ME3*	malic enzyme 3, NADP(+)-dependent, mitochondrial	pyruvate metabolic process, aerobic respiration, and oxidation–reduction process	Hi-L2165	1.20	1.60 × 10^–18^
			Hi-L2255	1.45	7.06 × 10^–27^
*PDK4*	pyruvate dehydrogenase kinase, isozyme 4	reactive oxygen species metabolic process and regulation of the lipid biosynthetic process	Hi-L2070	1.28	3.31 × 10^–7^
			Hi-L2255	1.03	9.66 × 10^–5^
*SLC24A3*	solute carrier family 47 (multidrug and toxin extrusion), member 1	cellular ion homeostasis	Hi-L2070	–1.50	0.02659149
			Hi-L2255	–1.32	0.035506
*SMPDL3A*	sphingomyelin phosphodiesterase, acid-like 3A	nucleoside triphosphate metabolic process	Hi-L2221	1.28	7.46 × 10^–13^
			Hi-L2255	1.27	5.98 × 10^–16^
*ABCA10*	ATP-binding, cassette, sub-family A, (ABC1), member, 10	lipid transporter activity	Hi-L2070	–1.85	0.04984293
*BPHL*	biphenyl hydrolase-like (serine hydrolase)	response to the xenobiotic stimulus	Hi-L2165	1.21	0.00245215
*BDH1*	3-hydroxybutyrate dehydrogenase, type 1	drug catabolic process	Hi-L2070	–1.56	3.95 × 10^–9^
*DYRK3*	dual-specificity, tyrosine-(Y)-phosphorylation, regulated, kinase, 3	cell cycle G2/M phase transition	Hi-L2221	4.49	4.05 × 10^–8^
*FBP1*	fructose-1,6-bisphosphatase 1	ATP metabolic process	Hi-L2221	1.94	6.13 × 10^–30^
*SUCNR1*	succinate receptor 1	myeloid cell activation involved in immune response	Hi-L2255	1.12	4.13 × 10^–7^
*TFRC*	transferrin receptor	cellular response to the drug, positive regulation of apoptotic signaling, viral life cycle, cellular cation homeostasis, and regulation of cell adhesion	Hi-L2255	1.04	4.42 × 10^–27^

a GO enrichment analysis and the profiling of DEGs involved in the modulation of THP-1 cells after infection with different *L. infantum* lines as described in Methods. The analysis was based on log_2_FC and FDRs. All genes presented in this list are statistically significant with an FDR value ≤ 0.05. Functions assigned correspond with GO sub-categories.

### Commonly Host-Modulated Genes by *L. infantum* Therapeutic Failure Clinical Isolates

**Figure 4:**
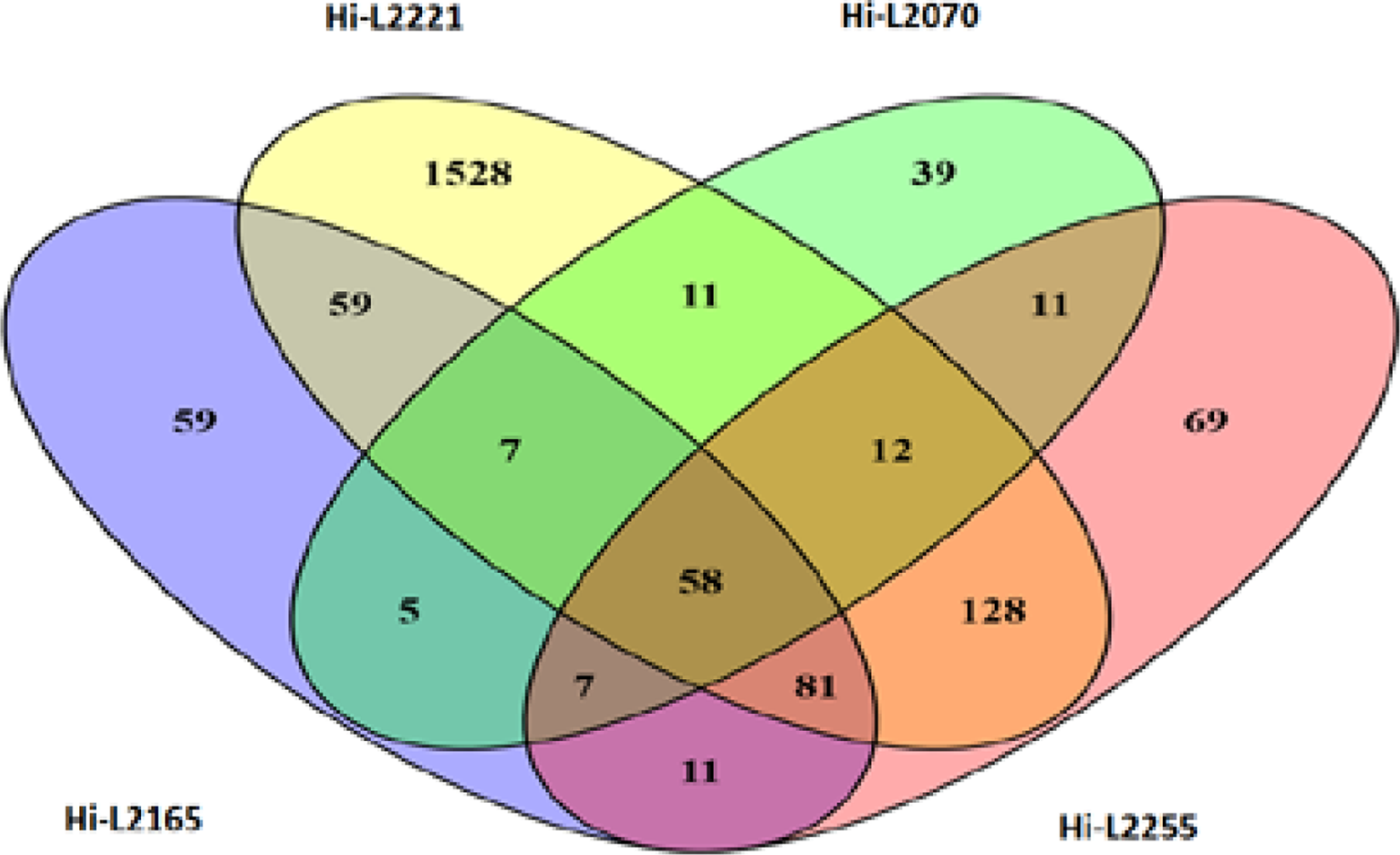
Venn diagram analysis of DEGs in THP-1 cells in response to infection with L2221, L2070, L2165, and L2255 *L. infantum* lines. Venn diagram of the total upregulated and downregulated DEGs with Log_2_FC ≥ 1 and Log_2_FC ≤ −1, respectively. The number of exclusive and common genes for each comparison are shown.

In addition to the previous transcriptomic analysis, Venn diagrams were performed in order to group the DEGs shared by the THP-1 cells infected with the four *L. infantum* lines, excluding the ones present in the host cells infected with reference line Hi-LJPC, uninfected cells, and cells infected with dead parasites as phagocytosis control in order to obtain a reduced and more specific list of DEGs involved in TF. We observed a total of 58 DEGs commonly modulated in Hi-L2221, Hi-L2070, Hi-L2165 and Hi-L2255 lines, including upregulated (log_2_FC ≥ 1) and downregulated (log_2_FC ≤ −1) genes (Figure 4). Some functional pathways and their genes of interest were the following: cellular and energy metabolism (*ACP5*, *CTSL*, *PCLB1*), cellular defense response (*CCL3*, *CCR2*, *COL4A1*, *HMOX1*, *PLCB1*, *SLC24A3*, *SMPDL3A*, *TNFRSF25*), lipid and cholesterol metabolism and transport (*ABCG1*, *ANO5*, *FABP5*) and transporter activity (*ABCG1*, *SLC1A3*, *SLC24A3*). Afterwards, from the above 58 DEGs commonly modulated in host cells infected with *L. infantum* lines, we selected four of these genes (*ABCG1*, *ANO5*, *COL4A1* and *HMOX1*) as relevant DEGs that could be involved in processes significant for the modulation of host cells in TF patients with leishmaniasis. Analysis of the modulation exerted by each independent line in comparison with the non-infected THP-1 cells revealed 1528 exclusive DEGs in cells infected by the *L. infantum* L2221 line, 39 DEGs in the case of the L2070 line, 59 DEGs for cells infected by the *L. infantum* L2165 line, and 69 DEGs in the case of L2255 (Figure 4).

### Analysis of Relevant Genes Selected for Validation

Taking into consideration the RNAseq analysis described in this article, we decided to select the most relevant DEGs for further validation, according to their possible contribution to the TF observed in patients infected with the different *Leishmania* lines. In this way, we classified them in four categories according to their function (Table 3). The first one grouped the genes *ABCG2* and *AQP9* involved in protection against xenobiotics and pathogens as well as transport across membranes. ATP-binding cassette transporters such as ABCG2 comprise a large superfamily of transmembrane proteins implicated in drug extrusion across membranes. They play a relevant role in the development of resistance, decreasing the intracellular concentration of drugs.^24,25^ In fact, the L2221 line showed resistance to Sb^III^ in the amastigote forms. In a similar way, changes in the expression of *AQP9* have been associated with drug resistance in malignant cells.^26^ Additionally, it transports glycerol, urea, and non-charged solutes, which can trigger the alteration of the osmotic permeability. Also, it has been proposed that one of the cellular responses following infection from different microorganisms includes an induction and recruitment of AQPs in order to promote the protection of microorganisms inside the cells.^27,28^

**Table 3:** Classification of THP-1 DEGs Selected for qPCR Validation^a^

Category	Gene ID	GO	THP-1 infected lines (log_2_FC)
Transporter activity, transmembrane movement of substances coupled to ATPase activity, and protection against xenobiotics and pathogens	*ABCG2*	GO:1990748	Hi-L2221 (2.12)
		GO:0055085	Hi-L2255 (1.33)
		GO:0042908	
	*AQP9*	GO:0006970	Hi-L2165 (1.40)
		GO:0015793	Hi-L2221 (2.43)
		GO:0015837	
		GO:0046942	
		GO:0007588	
Lipid and cholesterol metabolism and transport	*ABCG1*	GO:0033344	Hi-L2070 (1.01)
		GO:0033700	Hi-L2165 (1.84)
			Hi-L2221 (1.58)
			Hi-L2255 (1.87)
	*ANO5*	GO:1902476	Hi-L2070 (1.49)
		GO:0005229	Hi-L2165 (1.76)
			Hi-L2221 (1.29)
			Hi-L2255 (1.49)
	*CYP19A1*	GO:0016125	Hi-L2221 (2.78)
		GO:0006694	Hi-L2255 (1.67)
Cellular metabolism	*BDH1*	GO:0046951	Hi-L2070 (−1.56)
		GO:0046952	
	*DHDH*	GO:0005975	Hi-L2165 (2.72)
		GO:0022900	Hi-L2221 (3.15)
	*FBP1*	GO:0030388	Hi-L2221 (1.94)
		GO:0005975	
		GO:0006094	
		GO:0035690	
	*PDK4*	GO:0006006	Hi-L2070 (1.27)
		GO:0072593	Hi-L2255 (1.02)
Cellular defense response and signaling pathway	*COL4A1*	GO:0071230	Hi-L2070 (1.71)
		GO:0030198	Hi-L2165 (2.22)
			Hi-L2221 (3.01)
			Hi-L2255 (2.61)
	*HMOX1*	GO:0006879	Hi-L2070 (1.08)
		GO:0071243	Hi-L2165 (1.08)
			Hi-L2221 (1.56)
			Hi-L2255 (1.18)
	*SUCNR1*	GO:0002281	Hi-L2255 (1.11)
		GO:0007165	
		GO:0050729	
		GO:0050921	

a The list was prepared with relevant DEGs from the four main functional categories related with modulation of THP-1 cells infected with different clinical isolates of *L. infantum*. All genes presented in this list are statistical significant with an FDR value ≤ 0.05.

The next group of relevant biological processes was “lipid and cholesterol metabolism and transport”, including the genes *ABCG1*, *ANO5* and *CYP19A1*. A noticeable function of the ABC transporter ABCG1 is to manage the distribution of sterols from the endoplasmic reticulum.^29^ Additionally, some authors have exposed the implication of ABCG1 in the regulation of cholesterol efflux and cell autophagy in macrophages during infection of pathogens.^30^ As for ANO5, it is a calcium-activated chloride channel. Most anoctamins are phospholipid scramblases that facilitate the translocation of phospholipids between the cell membranes altering their physical properties.^31^ Another selected gene is *CYP19A1*, which catalyzes several reactions involved in the lipid metabolism and electron transport chain and plays an important role in the metabolism of xenobiotic substances.^32^

The third category established was “cellular metabolism”, comprising genes with different functions associated with pathogen infection of host cells and defense against drugs. For example, the *BDH1* gene plays an important role in the ketone body biosynthetic process and intracellular iron homeostasis. It has been described that endoplasmic reticulum stress and inflammation downregulate the expression of *BDH2* in human THP-1 macrophages.^33^ This appreciation is in accordance with our results since *BDH1* is downregulated in host cells infected with the L2070 line. Furthermore, we included in this group *DHDH* that belongs to the family of dihydrodiol dehydrogenases and is related to the metabolism of xenobiotics and sugars. It is considered by some authors as an anti-oxidation gene associated with drug resistance.^34^*FBP1* is another upregulated gene associated with cellular response to drugs and fructose metabolic processes. It has been hypothesized that FBP1 can cause the dysfunction of natural killer cells during lung cancer progression by weakening their glycolytic metabolism.^35^

Finally, we have included in the category of cellular metabolism the *PDK4* gene, which plays a key role in the regulation of the glucose and fatty acid metabolism. This gene has been established as a marker of enhanced fatty acid oxidation.^36^ Additionally, it has been described that PDK activity results in the production of several glycolytic intermediates that support pathogen replication through their employment in nucleotide synthesis.^37^

The last group of relevant biological processes was “cellular defense response and signaling pathway”, comprising *COL4A*, *HMOX1*, and *SUCNR1* genes. COL4A1 (collagen, type IV, alpha 1) plays a role in the generation of one of the components of type IV collagen, specifically the alpha 1 chain. Thus, it is crucial for the extracellular matrix organization and consequent interaction with nearby cells, allowing differentiation, survival, and establishment of infection.^38^ In fact, some authors have observed that collagen helps in the invasion and replication of some viruses.^39,40^ In the same line, it has been reported that expression of the extracellular matrix including laminin and collagens I, III, and IV is upregulated in HIV-1 infection.^41^ Also included in this category is HMOX1, which, like BDH1, it is involved in cellular iron homeostasis; additionally, it has been reported to be critical against oxidative stress.^42^ Interesting was that upregulation of *HMOX1* promotes persistence of *Leishmania* infection.^43^*SUCNR1*, as well in this category, encodes for a G-protein coupled receptor for succinate. According to some studies, the expression of *SUCNR1* is associated with the expression of chemokine and cytokine-related genes, and it is also involved in the infiltration of lymphocytes in ovarian cancer.^44^

### Validation of the DEGs by RT-qPCR

**Figure 5:**
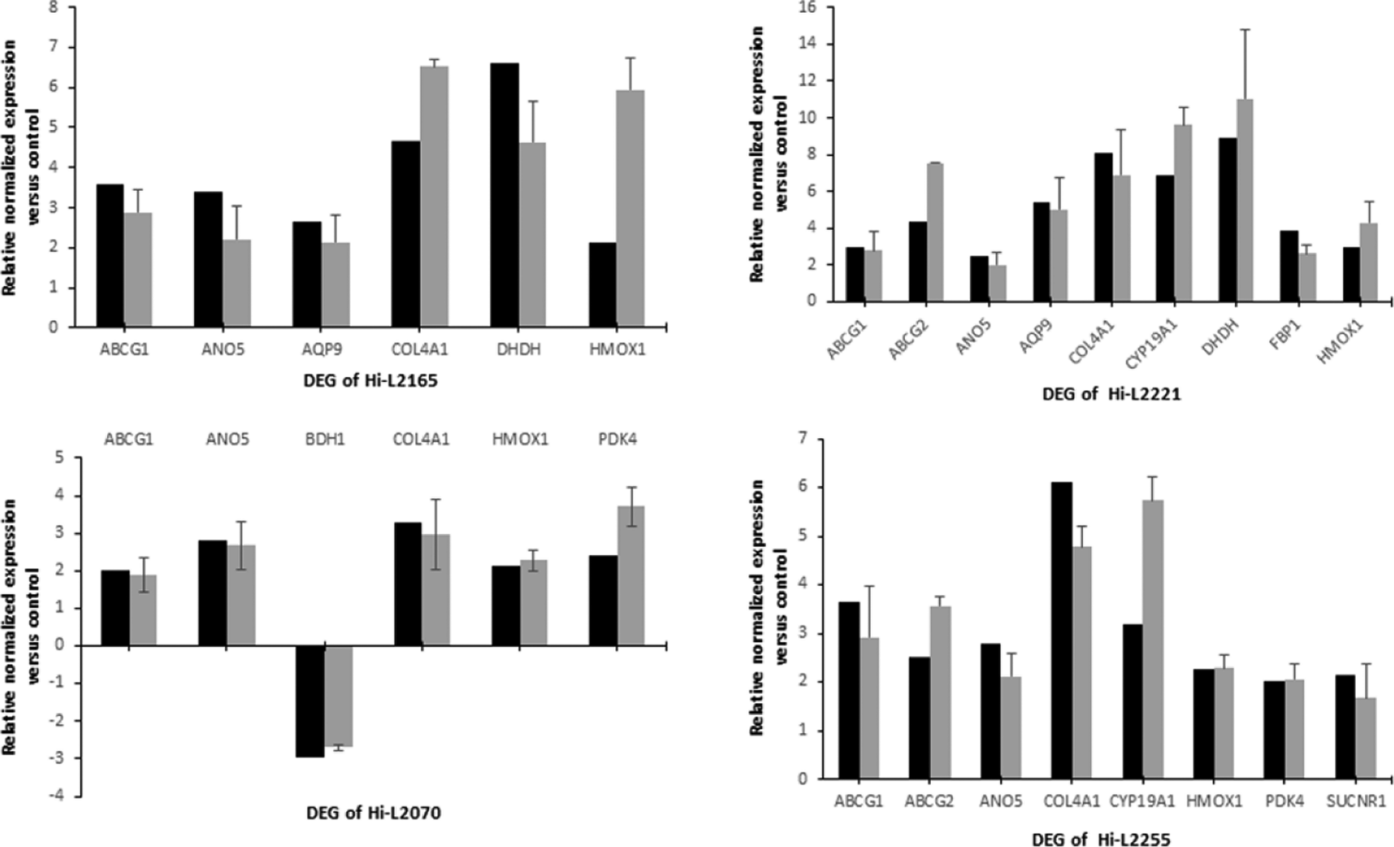
Comparative analysis of the relative expression levels of selected genes determined by RNA-seq (black) and validated by RT-qPCR (dark gray). The bars represent the mean ± SD values of fold-change expression of *ABCG1*, *ABCG2*, *ANO5*, *AQP9*, *BDH1*, *COL4A1*, *CYP19A1*, *DHDH*, *FBP1*, *HMOX1*, *PDK4* and *SUCNR1* determined from three independent biological replicates analyzed in triplicate. RT-qPCR expression values of the genes in each line were normalized with the expression of *ACTB*. The relative expression of each gene was calculated as the fold change between Hi-L2126, Hi-L2221, Hi-L2070, or Hi-L2255 and macrophages infected with heat-inactivated parasites (which was set to 1.0).

RT-qPCR validation assays were performed on 12 selected genes belonging to different functional categories (*ABCG1*, *ABCG2*, *ANO5*, *AQP9*, *BDH1*, *COL4A1*, *CYP19A1*, *DHDH*, *FBP1*, *HMOX1*, *PDK4* and *SUCNR1*) from the RNA-seq data in macrophages infected with different TF clinical isolates of *L. infantum*. To perform the validation, macrophages infected with heat-inactivated parasites and the actin gene *ACTB* were used as a control and as an internal standard, respectively. The reliability and accuracy of RNA-seq results were validated from the concordance between RNA-Seq and RT-qPCR data in comparative analysis (Figure 5).

## Conclusions

In this article, we demonstrate by RNA-Seq that different clinical *L. infantum* lines from TF patients promoted a different transcriptional response in THP-1 macrophages, which determine distinct behavior of the parasites that probably may account for parasite survival and TF of patients. Among relevant altered functional pathways and genes are the following: cellular and energy metabolism (*ACP5*, *CTSL*, *PCLB1*), cellular defense response (*CCL3*, *CCR2*, *COL4A1*, *HMOX1*, *PLCB1*, *SLC24A3*, *SMPDL3A*, *TNFRSF25*), lipid and cholesterol metabolism and transport (*ABCG1*, *ANO5*, *FABP5*) and transporter activity (*ABCG1*, *SLC1A3*, *SLC24A3*). These genes could be considered for a future rational therapeutic strategy for leishmaniasis that could include the combination of canonical anti-leishmanial compounds and host-directed therapy containing molecules interfering with some of these genes/proteins from host cells; this new therapeutic strategy could increase the efficacy of chemotherapy and the life of anti-parasitic drugs and will decrease the TF in patients with leishmaniasis.

## Methods

### Chemical Compounds

Amphotericin B (AmB), trivalent antimony (Sb^III^), paromomycin (PMM), Triton X-100, 4′,6-diamidino-2-phenylindole dilactate (DAPI), phorbol 12-myristate 13-acetate (PMA), resazurine, and sodium dodecyl sulfate (SDS) were purchased from Sigma-Aldrich (St. Louis, MO). Miltefosine (Mil) was purchased from Zentaris GmbH (Frankfurt, Germany). l-Glutamine and penicillin/streptomycin were obtained from Gibco. All chemicals were of the highest quality available.

### Culture of *L. infantum* Lines and THP-1 Cells

We used promastigotes of *L. infantum* lines: (i) JPC-M5 (MCAN/ES/98/LLM-877) (LJPC) as a genomic reference line and (ii) LLM2070, LLM2165, LLM2255, and LLM2221 lines (L2070, L2165, L2255, and L2221), isolated from TF HIV patients with VL and unsuccessfully treated with liposomal AmB (from the WHO Collaborating Center for Leishmaniasis, Instituto de Salud Carlos III; Dr. F. Javier Moreno). All these *L. infantum* lines were grown at 28 °C in the RPMI 1640-modified medium (Invitrogen) supplemented with 10% hiFBS (Invitrogen), as described.^45^ Human myelomonocytic cells THP-1 were grown at 37 °C and 5% CO_2_ in the RPMI-1640 medium supplemented with 10% hiFBS, 2 mM glutamate, 100 U/mL penicillin, and 100 mg/mL streptomycin, as described.^46^

### *In Vitro* Macrophage Infection

First, THP-1 cells were plated in 25 cm^2^ flasks at a ratio of 3 × 10^6^ cells or 5 × 10^5^ cells/well in 24-well plates. Then, cells were differentiated to macrophages with 20 ng/mL of PMA treatment for 48 h. Macrophage-differentiated THP-1 cells were infected with different *L. infantum* promastigotes incubated for 72 h in an acid medium plus 10% hiFBS, optimizing their infection ability, or heat-killed promastigotes (LJPC incubated for 1 h at 65 °C). The macrophage/parasite ratio was 1:10 as described previously.^45^ After 24 h infection at 35 °C and 5% CO_2_ in the RPMI 1640 medium plus 5% hiFBS, extracellular parasites were removed by washing three times with PBS (1.2 mM KH_2_PO_4_, 8.1 mM Na_2_HPO_4_, 130 mM NaCl, 2.6 mM KCl, pH 7) and infected macrophages were maintained in the RPMI 1640 medium plus 10% hiFBS at 37 °C and 5% CO_2_ for 96 h. Finally, the samples were collected in Qiazol (Qiagen), and total RNA was isolated in the Genomic Unit of GENyO facilities (Granada, Spain). Additionally, to determine the parameters related with infectivity such as the percentage of infection or the number of amastigotes by macrophages and in parallel with the infection assay destined for RNA extraction, macrophages were infected with the same *L. infantum* lines and treated under the same conditions. Briefly, cells were fixed for 30 min at 4 °C with 2.5% paraformaldehyde in PBS and permeabilized with 0.1% Triton X-100 in PBS for 30 min. Intracellular parasites were nuclear-stained with DAPI (Invitrogen) and detected by fluorescence microscopy. Additionally, we used as controls macrophages that had been allowed to phagocytose heat-killed parasites and uninfected macrophages. In this way, we will identify changes in gene expression that represent a general result of phagocytosis and phagolysosome formation rather than being specific to *Leishmania* infection. The abbreviations for different infected host cell lines used in this work are the following: (i) Hi-LJPC for THP-1 cells infected with the *L. infantum* LJPC line, (ii) Hi-L2070 for THP-1 cells infected with the *L. infantum* LLM-2070 line, (iii) Hi-L2165 for THP-1 cells infected with the *L. infantum* LLM-2165 line, (iv) Hi-L2255 for THP-1 cells infected with the *L. infantum* LLM-2255 line, and (v) Hi-L2221 for THP-1 cells infected with the *L. infantum* LLM-2221 line.

### Drug Sensitivity Analysis in Intracellular Amastigotes of *L. infantum* Lines

Macrophage-differentiated THP-1 cells were plated at a density of 3 × 10^4^ macrophages/well in 96-well plates, infected at a macrophage/parasite ratio of 1:10 with stationary-phase promastigotes of *L. infantum* isolates, and incubated at 35 °C and 5% CO_2_ in the RPMI 1640 medium plus 5% hiFBS. 24 h after infection, extracellular parasites were removed by washing three times with PBS buffer. Then, infected macrophages were incubated for 72 h in the RPMI 1640 medium plus 10% hiFBS at 37 °C and 5% CO_2_ atmosphere, with different concentrations of anti-leishmanial compounds indicated in the text. To determine the susceptibility of *L. infantum* amastigotes, infected macrophages maintained in 96-well plates were lysed as previously described.^47^ Finally, the EC_50_ was determined using the resazurine colorimetric assay.

### RNA Isolation and cDNA Library Preparation

RNA samples were extracted using a QIAamp RNA miRNeasy Micro Kit (Qiagen/Qiacube). Three independent biological replicates of the cells infected with different *Leishmania* lines and controls were collected. Then, samples were treated with a DNase kit and quantified using a Nanodrop One (Thermo Fisher). Quality and quantity checks of RNA samples were performed using a 2100 Bioanalyzer (Agilent Technologies). RNA integrity was evaluated using an *Agilent 2100 Bioanalyzer* system with an *RNA 6000 Nano Lab Chip* kit (Agilent Technologies). Poly(A)-enriched cDNA libraries were generated using a TruSeq Stranded mRNA kit (Illumina) and checked for quality and quantity using a 2100 Bioanalyzer and quantitative PCR. All these analyses and sequencing were performed by GENyO facilities (Granada, Spain).

### RNA-Seq Data Generation and Pre-processing

The next-generation sequencing run for whole transcriptome sequencing was performed using the paired-end (PE) 2 × 75 bp library on the NextSeq 500 (Illumina, San Diego, CA, USA) platform at GENyO (Granada, Spain) using High Output Kit v2.5 (150 cycles), which generated 14.5 million homogenized reads per sample. Raw data were generated for each of the libraries from the three samples (Table S1). The RNA-seq data are available at NCBI Short Read Archive (SRA) under accession number PRJNA781438.

### Data Analysis

For the purpose of analyzing transcriptomic samples, the miARma-Seq pipeline was used.^48^ This workflow achieves all steps from raw data to the calculation of DEGs. In the first place, the raw data were evaluated with FastQC software to analyze the quality of reads.^49^ Subsequently, after sample filtering by quality and homogenization of the number of reads per sample using Seqtk software^50^ (on average 6.8 million), miARma-Seq aligns all sequences using HISAT2,^51^ resulting in a 80% of properly aligned reads, ranging from 40 to 90% depending on the infection rate. With this aim, we have the protein coding genes from the *Homo sapiens* Gencode version M26 genome-build: Homo_sapiens. GRCh38 was used as a reference genome.

### Differential Expression Analysis

In order to carry out the differential expression analysis, the edgeR package was used.^52,53^ Low-expressed genes were removed, and the remaining genes were normalized by the trimmed mean of *M*-values (TMM) method.^52^ Furthermore, we calculated reads per kilobase per million mapped reads (RPKM) and counts per million (CPM) and log_2_-counts per million (log-CPM) per gene on each sample.^50^ In order to determine the replicability of the samples, principal component analysis and hierarchical clustering of normalized samples were used to obtain an overview of the similarity of RNA-sequencing samples.^53−55^ After that, all samples were analyzed as we do not identify any clear outliers. DEGs comparing the three replicates of TF lines adjusted by control samples were calculated. All genes having an FDR value ≤ 0.05 and a fold change ≥ 2 were marked as DEGs. Log_2_FC was used to evaluate the significance and the change in expression of a gene respectively between different types of samples.

### Enrichment Analysis

The clusterProfiler Bioconductor package^56^ was used with the aim of identifying differential gene expression effects by carrying out a functional enrichment study. For this purpose, DEGs were compared against all expressed genes in the RNA-seq assay and we obtained GO terms from the Bioconductor *H. sapiens* database and associated to Entrez gene identifiers in an *orgDB* R object through the *AnnotationForge* package to be used with *clusterProfiler*. Therefore, GO enrichment analysis was calculated for Biological Process.

### RT-qPCR Validation

RT-qPCR validation assays were performed using total RNA isolated for a set of selected genes. Reverse transcription was performed using 2 μg of total RNA with a qScript cDNA Synthesis Kit (Quanta Biosciences, Inc.), according to the manufacturer’s instructions. The Primer3 software^57^ was used to design specific primer pairs, which were utilized to amplify cDNA (Table S2). The efficiency of each primer was determined using standard curves performed with 2-fold serial dilutions of the synthetized cDNA. A CFX96 cycler (BioRad) was used to carry out qPCR analysis, as described previously.^58^ Data were normalized by actin gene *ACTB* expression and relative to the control sample (heat-inactivated parasites) using the CFX Manager software with the ΔΔ*Ct* method.^59,60^

## Supporting Information Available

RNA-seq reads of THP-1 cells and primer sequences for RT-qPCR (PDF)
