# Supplementary material for: Transcriptomic Analysis in Human Macrophages Infected with Therapeutic Failure Clinical Isolates of Leishmania infantum
Source: ACS Infect Dis. 2022 Mar 30;8(4):800–10. doi: 10.1021/acsinfecdis.1c00513 (PMC9003231; doi:10.1021/acsinfecdis.1c00513)
Supplement: Supplementary file 1 — id1c00513_si_001.pdf [file id1c00513_si_001.pdf]

# Supporting Information

Transcriptomic analysis in human macrophages infected with therapeutic failure clinical isolates of *Leishmania infantum*

Ana Perea-Martínez[a], Raquel García-Hernández[b], José Ignacio Manzano[c], Francisco Gamarro[d].

*Instituto de Parasitología y Biomedicina "López-Neyra", IPBLN-CSIC, Parque Tecnológico de Ciencias de la Salud, Avda. del Conocimiento s/n, 18016 Armilla, Granada, Spain.*

[a]*anapereama@ipb.csic.es*. [b]*raquelg@ipb.csic.es*. [c]*nacho.manzano@ipb.csic.es*.
[d] *gamarro@ipb.csic.es*

*Table of Contents*

**Table S1. RNAseq reads of THP-1 cells** **S2**

**Table S2. Primer sequences for RT-qPCR** **S3**

**Table S1. RNAseq reads of THP-1 cells**

| Sample name | Initial reads[a] | Homogenized Reads[b] | Aligned Reads[c] | Final reads[d] | Spearman correlation | | |
|---|---|---|---|---|---|---|---|
| | | | | | Rho R1_R2 | Rho R1_R3 | Rho R2_R3 |
| Hi-L2165 R1 | 37305366 | 14640356 | 10449564 (71.38%)[e] | 4617423 | | | |
| Hi-L2165 R2 | 36357116 | 14080836 | 10437034 (74.12%) | 4622659 | 0.8504 | 0.8521 | 0.8492 |
| Hi-L2165 R3 | 40279450 | 14587072 | 10438894 (71.56%) | 4618914 | | | |
| Hi-L2221 R1 | 15766016 | 15766016 | 8377808 (53.14%) | 3822637 | | | |
| Hi-L2221 R2 | 15007286 | 14217352 | 8338666 (58.65%) | 3822006 | 0.8485 | 0.8491 | 0.8501 |
| Hi-L2221 R3 | 15183074 | 15183074 | 7933198 (52.25%) | 3610876 | | | |
| Hi-LJPC R1 | 70161332 | 12851868 | 10494162 (81.65%) | 4626503 | | | |
| Hi-LJPC R2 | 71533386 | 12514220 | 10468334 (83.65%) | 4622522 | 0.8422 | 0.8365 | 0.8345 |
| Hi-LJPC R3 | 64492256 | 12454024 | 10354376 (83.14%) | 4607134 | | | |
| Hi-L2070 R1 | 36183688 | 14474940 | 10615282 (73.34%) | 4631126 | | | |
| Hi-L2070 R2 | 36810964 | 14569862 | 14569862 (72.43%) | 4627814 | 0.8501 | 0.8509 | 0.8504 |
| Hi-L2070 R3 | 38067316 | 15030990 | 10582324 (70.40%) | 4623885 | | | |
| Hi-L2255 R1 | 15766016 | 14563422 | 10767024 (72.31%) | 4631672 | | | |
| Hi-L2255 R2 | 15007286 | 15186194 | 10384472 (68.38%) | 4613996 | 0.8512 | 0.848 | 0.8507 |
| Hi-L2255 R3 | 15183074 | 14296376 | 10342598 (72.34%) | 4618494 | | | |
| Phagocytosis control R1 | 14155514 | 12354592 | 10767024 (87.15%) | 4632581 | | | |
| Phagocytosis control R2 | 13873380 | 12485250 | 10876472 (87.11%) | 4637532 | 0.8443 | 0.8444 | 0.8436 |
| Phagocytosis control R3 | 11808612 | 11808612 | 10758070 (91.10%) | 4635661 | | | |
| Uninfected cells R1 | 16249142 | 12086856 | 10886286 (90.07%) | 4637856 | | | |
| Uninfected cells R2 | 16181098 | 11665374 | 10894640 (93.39%) | 4637532 | 0.8458 | 0.8457 | 0.8462 |
| Uninfected cells R3 | 15900940 | 12007916 | 10948074 (91.17%) | 4637311 | | | |

[a]Reads obtained directly from the sequencer. [b]Reads randomly selected by seqtk to produces scaled reads files. [c]Reads aligned to human genome. [d]Aligned reads quantified in coding exons. [e]Percentage of reads aligned to human genome.

**Table S2. Primer sequences for RT-qPCR**

| Genes | | Primer Sequences (5'→3') |
|---|---|---|
| *ABCG1* | F | AGGAGAAGGATGAAGGCAGA |
| | R | GAAGAACATGACTGGAGGGT |
| *ABCG2* | F | TTTATCCGTGGTGTGTCTGG |
| | R | TTGTTCGTCCCTGCTTAGA |
| *ANO5* | F | AGAACCTCCCAATCCTACCA |
| | R | TCAAGTCTAAAGGCTGCTCC |
| *AQP9* | F | AGTTCTTGGGAGCCTTTGTG |
| | R | TCGCCAGAGATAGATACGGAG |
| *BDH1* | F | CGTCGTCAATATCAGCAGCA |
| | R | TGTGACAGCATCGATGACAG |
| *COL4A1* | F | TCCCTGGTGAAAGAGGAGAA |
| | R | GCGGCTGACATTCCACAAT |
| *CYP19A1* | F | GTGACCAATGAATCGGGCTA |
| | R | TTTGATGAGGAGAGCTTGCC |
| *DHDH* | F | CATCGGCATCTACTGTGTCC |
| | R | AGTGTCATCCACACCTGTTTC |
| *FBP1* | F | GCTGAGACTGCTGTACGAAT |
| | R | CTCTGGTGAATGTCTGTGGG |
| *HMOX1* | F | AAAGATTGCCCAGAAAGCCC |
| | R | GCTGAGTGTAAGGACCCATC |
| *PDK4* | F | TTAAGAATGCAATGCGGGC |
| | R | ACCACCTCCTCTGTCTGAA |
| *SUCNR1* | F | AAAGCAGAGGAATAGGCAGG |
| | R | AAAGCACAGAGAAGATTACCAC |
| *ACTB* | F | ATTGCCGACAGGATGCAGAA |
| | R | GCTGATCCACATCTGCTGGAA |

RT-qPCR, Reverse transcriptase quantitative PCR.; F, Forward; R, Reverse
